# Is Paromomycin an Effective and Safe Treatment against Cutaneous Leishmaniasis? A Meta-Analysis of 14 Randomized Controlled Trials

**DOI:** 10.1371/journal.pntd.0000381

**Published:** 2009-02-17

**Authors:** Dae Hyun Kim, Hye Jin Chung, Joachim Bleys, Reza F. Ghohestani

**Affiliations:** 1 Department of Medicine, Beth Israel Deaconess Medical Center, Boston, Massachusetts, United States of America; 2 Department of Dermatology and Cutaneous Biology, Jefferson Medical College, Philadelphia, Pennsylvania, United States of America; 3 Welch Center for Prevention, Epidemiology, and Clinical Research, The Johns Hopkins Medical Institutions, Baltimore, Maryland, United States of America; 4 Division of Dermatology and Cutaneous Surgery, Department of Medicine, University of Texas Medical School at San Antonio, Texas, United States of America; 5 Texas Dermatology Institute, San Antonio, Texas, United States of America; Drugs for Neglected Diseases Initiative, Switzerland

## Abstract

**Background:**

High cost, poor compliance, and systemic toxicity have limited the use of pentavalent antimony compounds (SbV), the treatment of choice for cutaneous leishmaniasis (CL). Paromomycin (PR) has been developed as an alternative to SbV, but existing data are conflicting.

**Methodology/Principal Findings:**

We searched PubMed, Scopus, and Cochrane Central Register of Controlled Trials, without language restriction, through August 2007, to identify randomized controlled trials that compared the efficacy or safety between PR and placebo or SbV. Primary outcome was clinical cure, defined as complete healing, disappearance, or reepithelialization of all lesions. Data were extracted independently by two investigators, and pooled using a random-effects model. Fourteen trials including 1,221 patients were included. In placebo-controlled trials, topical PR appeared to have therapeutic activity against the old world and new world CL, with increased local reactions, when used with methylbenzethonium chloride (MBCL) compared to when used alone (risk ratio [RR] for clinical cure, 2.58 versus 1.01: RR for local reactions, 1.60 versus 1.07). In SbV-controlled trials, the efficacy of topical PR was not significantly different from that of intralesional SbV in the old world CL (RR, 0.70; 95% confidence interval, 0.26–1.89), whereas topical PR was inferior to parenteral SbV in treating the new world CL (0.67; 0.54–0.82). No significant difference in efficacy was found between parenteral PR and parenteral SbV in the new world CL (0.88; 0.56–1.38). Systemic side effects were fewer with topical or parenteral PR than parenteral SbV.

**Conclusions/Significance:**

Topical PR with MBCL could be a therapeutic alternative to SbV in selected cases of the old world CL. Development of new formulations with better efficacy and tolerability remains to be an area of future research.

## Author Summary

Millions of people worldwide are suffering from cutaneous leishmaniasis that is caused by parasites of the genus *Leishmania*. Although pentavalent antimony compounds are the treatment of choice, their use is limited by high cost, poor compliance, and systemic toxicity. Paromomycin was developed to overcome such limitations. However, there is no consensus on its efficacy. This meta-analysis assessed the efficacy and safety of paromomycin compared with placebo and pentavalent antimony compounds. Fourteen randomized controlled trials, including 1,221 patients, met our selection criteria. Topical paromomycin appeared to have therapeutic activity against the old world and new world cutaneous leishmaniasis, with increased local reactions, when combined with methylbenzethonium chloride. Topical paromomycin was not significantly different from intralesional pentavalent antimony compounds in treating the old world form, whereas it was inferior to parenteral pentavalent antimony compounds in treating the new world form. However, a similar efficacy was found between parenteral paromomycin and pentavalent antimony compounds in treating the new world form. Fewer systemic side effects were observed with topical and parenteral paromomycin than pentavalent antimony compounds. These results suggest that topical paromomycin with methylbenzethonium chloride could be a therapeutic alternative to pentavalent antimony compounds for selected cases of the old world cutaneous leishmaniasis.

## Introduction

More than 12 million people in 88 countries suffer from leishmaniasis, a condition caused by parasites of the genus *Leishmania* [1]. Annually, two million new cases of leishmaniasis are diagnosed, of which about one quarter present as visceral leishmaniasis, a potentially fatal condition. The rest present as cutaneous leishmaniasis (CL), a non-fatal yet severely disfiguring condition characterized by skin lesions and unsightly scars on the face and extremities. Over the past decade, the worldwide prevalence and geographical distribution of CL have expanded.

Pentavalent antimony compounds (SbV), such as sodium stibogluconate (SB) or meglumine antimoniate (MA), have been the mainstay of the treatments [2]. Despite its efficacy, SbV is limited by high cost, poor compliance due to a prolonged course of intramuscular or intravenous injections, and potentially reversible systemic toxicity [3]–[6]. Resistance is also of particular concern [7]. Among various species causing the old world and new world CL, certain species are more likely to self-cure at a slower rate or progress to diffuse or mucocutaneous form than others [8]. Due to such clinical significance, the treatment has been mainly in the form of topical application in the old world CL and systemic in the new world CL. Seeking an alternative to SbV for localized CL has been of particular interest over the past decades.

Therapeutic activity of paromomycin (synonymous with aminosidine) (PR) was first reported in the 1960's [9],[10]. In the 1980's, El-On et al. demonstrated therapeutic activity of PR in an *in vitro* study [11]. Epicutaneous administration of PR (topical PR, hereafter) with 12% MBCL (“first-generation formulation”) further showed promising results in animal [12] and human studies [13]. In the early 1990's, MBCL was replaced with urea to reduce local side effects from MBCL (“second-generation formulation”) [14],[15]. Even though PR has also been administered parenterally [4],[5], topical PR, in particular, has several advantages over SbV, because of its fewer systemic side effects, lower cost, and convenience [3],[16],[17]. Thus, it could be a good therapeutic alternative to SbV. However, clinical trials of topical PR and parenteral PR have showed widely varying results on the efficacy and safety in treating CL. Its cure rate ranges from 4% [18] to 93% [4] and its efficacy compared with SbV has been equivocal [19]. Therefore, we performed a systematic review and meta-analysis to assess the efficacy and safety of various PR regimens as compared to placebo and SbV.

## Methods

### Data sources and study selection

We searched PubMed, Scopus, and Cochrane Central Register of Controlled Trials, with no language restriction, from inception through August 2007, to identify all randomized controlled trials evaluating the efficacy and safety of PR for CL, using the following search terms: *cutaneous leishmaniasis*, *paromomycin*, *aminosidine*, and *randomized controlled trials*. Detailed strategies are described in Appendix S1. The initial search was complemented by a manual search of the reference lists from the retrieved articles and the “Related Articles” function of PubMed. Because various types of PR and SbV regimens were tested, we tried to make clinically meaningful comparisons by pooling the data within similar groups of trials.

Reports were excluded according to the following *a priori* criteria: 1) reviews, meta-analyses, or editorials; 2) case reports or retrospective studies; 3) animal or *in-vitro* studies; 4) no randomized control group; and 5) no data on efficacy or safety outcomes of PR treatment. When a study originated several reports [5],[20], the report with the largest sample size or the longest follow-up was included [5]. We further excluded two trials that compared different duration or dose of PR without placebo or SbV control group [21],[22]; one trial that compared different MA regimens as an augmentation of the same topical PR regimen [23]; two trials that randomized lesions instead of patients [18],[24] (because certain local treatments may lead to improvement in untreated lesions in the same individual [25],[26]); and four trials that compared PR with second-line treatments such as pentamidine [4], ketoconazole [27], and photodynamic therapy [24],[28]. All the retrieved reports were independently reviewed by two investigators (HJC and DHK) for eligibility and any disagreements were resolved by consensus.

### Data extraction and validity assessment

Two investigators (HJC and DHK) independently extracted data on participants' characteristics, predominant parasite species, interventions, and outcomes from included reports by using a standardized data collection form. When the parasite species were not reported, we assumed that it was the same as in other trials conducted in the same geographical region [17],[27],[29]. We evaluated the quality of studies using the following criteria: 1) double-blind; 2) concealment of treatment allocation; 3) blinding of outcome assessment; and 4) intention-to-treat analysis. Concealment of treatment allocation was adequate if patients and enrolling investigators could not predict assignment. Outcome assessment was blinded if the investigator who assessed the outcome had no knowledge of treatment assignment. The analysis was performed according to the intention-to-treat principle if all randomized patients were included in the analysis and kept in the originally assigned groups. If there was not enough information to assess the quality, it was assumed inadequate. Any disagreements were resolved by consensus.

The main outcome was clinical cure, defined as complete healing, disappearance, or reepithelialization of all lesions. The secondary outcome was clinical improvement, defined as complete or incomplete healing or reepithelialization of the lesion or any reduction in the size. In addition, local and systemic side effects were assessed. Local side effects included pain, burning sensation, pruritus, erythema, edema, and inflammation at the administration site. Systemic side effects included myalgia, generalized symptoms (i.e. fever, malaise, weakness, and anorexia), headache, arthralgia, generalized eruptions, and laboratory abnormalities on blood counts, chemistry, and liver function tests.

### Quantitative data synthesis

Trials with placebo control group were analyzed separately from trials with SbV control group. Pooled estimates and 95% confidence intervals (CIs) of risk ratios (RRs) were calculated by using an inverse-variance weighted random-effects model [30] according to the intention-to-treat principle. Between-study heterogeneity was quantified using the χ^2^ and *I^2^* statistics [31]. A random-effects meta-regression analysis was performed to evaluate whether the heterogeneity among trials with placebo control group was explained by duration of lesion, length of PR treatment, and type of PR regimen. Duration of lesion and length of PR treatment have been proposed as potential explanations for inconsistent results in previous studies [27],[32]. Although different species or type of SbV regimen could have contributed to the heterogeneity, we were not able to examine such factors due to an insufficient number of trials. In addition, the influence of the study quality criteria was evaluated. We chose this approach rather than excluding trials based on a composite quality scale, because disagreement between different scales is common and valuable information may get excluded by the latter approach. We conducted sensitivity analysis by examining the relative influence of each study on the pooled estimate by excluding one study at a time. Finally, a publication bias was examined by the Begg's test (rank correlation method) and Egger's test (weighted regression). Statistical significance was defined as P<0.05 and all statistical analyses were conducted with Stata/SE version 9.2 (StataCorp, College Station, TX).

## Results

### Trial flow and study characteristics

**Figure 1:**
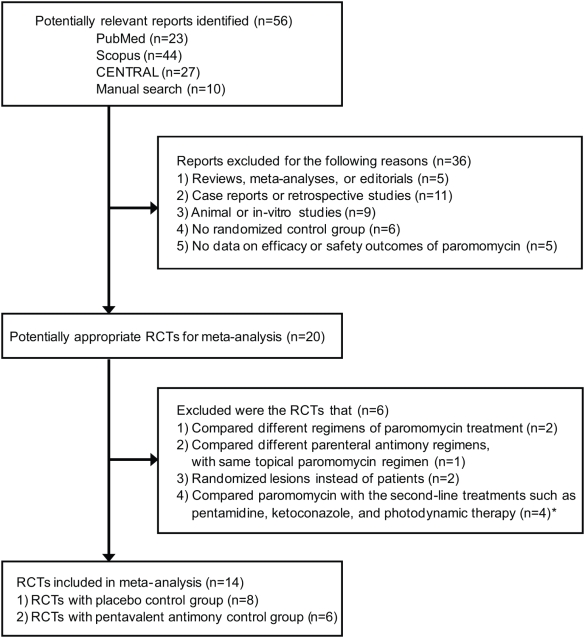
Flow Diagram of the Study Selection Process. Abbreviations: CENTRAL, Cochrane Central Register of Controlled Trials; RCT, randomized controlled trials. * When a trial involved a second-line treatment as well as placebo or pentavalent antimony compounds, the data on placebo [28] or pentavalent antimony compounds [4] were included.

Fourteen randomized controlled trials with a total of 1,221 patients satisfied our selection criteria [3]–[6], [16], [17], [28], [29], [32]–[37]. The study selection process was summarized in Figure 1.

Table 1 summarizes the study characteristics. Eight trials [17], [28], [29], [32]–[34],[36],[37] were conducted in the Middle East and North Africa where CL was caused by *L.major* (old world CL), and six trials [3]–[6],[16],[35] were conducted in Central and South America where CL was caused by *L.braziliensis*, *L.panamensis*, and *L.chagasi* (new world CL). The mean age ranged from 5 to 24 years and the proportion of male varied between 42% and 100%. The average duration of lesions ranged from 15 to 105 days. Four types of PR regimen were evaluated: topical PR alone [17], [29], [32]–[36]; topical PR with MBCL [3],[6],[16],[28],[37]; topical PR with MBCL and parenteral MA [6]; and parenteral PR [4],[5]. Ointment was used for all topical formulations, except for one trial [29] where a lotion form was used. Three trials [16],[28],[37] used MBCL as a vehicle, whereas four [29],[32],[34],[35] used urea and one [36] used a paraffin and wool fat vehicle. Among SbV-controlled trials, two trials [17],[33] used intralesional regimen and four [3]–[6] used parenteral regimen. After a follow-up period of 27 to 455 days, the efficacy was assessed clinically [3],[6],[16],[17],[35] or in combination with parasitological examination [4], [5], [28], [29], [32]–[34],[36],[37]. Seven [16],[28],[29],[32],[34],[35],[37] were double-blinded and two [3],[6] were only double-blinded with respect to topical treatment.

**Table 1:** Randomized Controlled Trials of Paromomycin in Cutaneous Leishmaniasis.

Author, Year (Country)	Age (yr)	Male (%)	Predominant Parasite	Duration of Lesion (d)	Double-Blind	Follow-up* (d)	Paromomycin Group		Control Group		Assessed Outcome
							Regimen†	*n* ‡	Regimen†	*n* ‡	
***Randomized Controlled Trials with Placebo Control Group (n = 8)***											
El-Safi, 1990 [36] (Sudan)	NR	NR	*L.major*	NR	No	30	15% PR/paraffin and wool fat, 10 *d*	16 (20)	Placebo, 10 *d*	15 (20)	C, P
El-On, 1992 [37] (Israel)	23	72	*L.major*	105	Yes	60	15% PR/(12 or 5% MBCL), 10–20 *d*	39 (NR)	Placebo, 10–20 *d*	15 (NR)	C, P
Salah, 1995 [34] (Tunisia)	19	51	*L.major*	NR	Yes	105	15% PR/10% urea, 14 *d*	52 (NR)	Placebo, 14 *d*	56 (NR)	C, P
Asilian, 1995 [32] (Iran)	5·5∥	52	*L.major*	15∥	Yes	105	15% PR/10% urea, 14 *d*	118 (126)	Placebo, 14 *d*	116 (125)	C, P
Neva, 1997 [35] (Honduras)	NR	NR	*L.chagasi*	NR	Yes	133	15% PR/10% urea, 28 *d*	23 (23)	Placebo, 28 *d*	30 (30)	C
Arana, 2001 [16] (Guatemala)	21	NR	*L.braziliensis*	103	Yes	384	15% PR/12% MBCL, 20 *d*	35 (38)	Placebo, 20 *d*	33 (38)	C
Iraji, 2005 [29] (Iran)	21	51	*L.major* **	51	Yes	60	15% PR/10% urea, 30 *d*	30 (40)	Placebo, 30 *d*	35 (40)	C, P
Asilian, 2006 [28] (Iran)	23	46	*L.major*	36	Yes	90	15% PR/12% MBCL, 28 *d*	34 (35)	Placebo, 28 *d*	30 (33)	C, P
***Randomized Controlled Trials with Pentavalent Antimony Control Group (n = 6)***											
Hepburn, 1994 [5] (UK/Belize§)	24	NR	*L.braziliensis L.mexicana*	93	No	362	PR 14 mg/kg IV, 20 *d*	17 (17)	SB 20 mg/kg IV, 20 *d*	17 (17)	C, P
Correira, 1996 [4] (Brazil)	NR	NR	*L.braziliensis*	47	No	365	PR 20 mg/kg IM, 20 *d*	15 (15)	MA 10 mg/kg IM, 20 *d*	16 (16)	C, P
Soto, 1998 [6] (Colombia)	NR	NR	*L.panamensis L.braziliensis*	NR	No††	270–360	15% PR/12% MBCL, 10 *d*+MA 20 mg/kg IM, 3 or 7 *d*	89 (89)	(Placebo, 10 *d*+MA 20 mg/kg IM, 7 *d*) or MA 20 mg/kg IM, 20 *d*	61 (61)	C
Faghihi, 2003 [17] (Iran)	16	42	*L.major* **	NR	No	455	15% PR/10% urea, 45 *d*	48 (48)	MA 5 ml IL, 1/*wk*, 35 *d*	48 (48)	C
Armijos, 2004 [3] (Ecuador)	20	NR	*Viannia subgenus*	88	No††	364	15% PR/(12% MBCL or 10% urea), 30 *d*	59 (80)	MA 20 mg/kg IM, 10 *d*	36 (40)	C
Shazad, 2005 [33] (Iran)	21	100	*L.major*	38	No	27	15% PR/10% urea, 20 *d*	29 (30)	MA 1 ml IL, every other day, 20 *d*	27 (30)	C, P

Abbreviations: yr, year; d, day; NR, not reported; PR, paromomycin; C, clinical response; P, parasitological response; MBCL, methylbenzethonium chloride; IV, intravenous; SB, sodium stibogluconate; IM, intramuscular; PO, per os; MA, meglumine antimoniate; IL, intralesional.

*: The duration of follow-up included treatment period.

**†:** The administration route is topical unless specified otherwise.

**‡:** The number of patients or lesions who were originally assigned to each treatment was displayed in parenthesis.

**§:** Participants were British soldiers who contracted cutaneous leishmaniasis in Belize.

**∥:** When the mean was not reported, the median was presented.

**: When the parasite species was not reported, it was assumed to be the same as in other trials conducted in the same geographical region.

**††:** Studies were only double-blinded with respect to topical treatment.

### Efficacy of PR versus placebo

**Figure 2:**
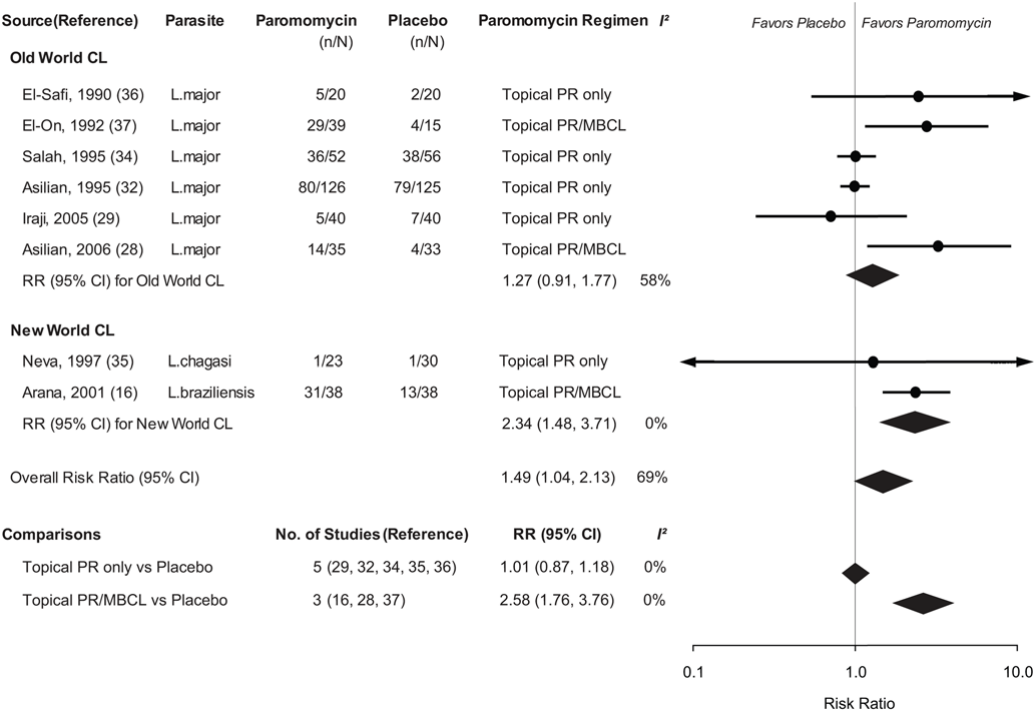
Meta-analysis of the Efficacy of Paromomycin Compared with Placebo*. Abbreviations: CI, confidence interval; CL, cutaneous leishmaniasis; MBCL, methylbenzethonium chloride; PR, paromomycin; RR, risk ratio. * Pooled RRs and 95% CIs of clinical cure were calculated using an inverse-variance weighted random-effects model and displayed in diamonds in the figure. The *I^2^* statistic describes the percentage of total variation across the studies that is attributable to heterogeneity rather than chance.

Absolute rate of clinical cure comparing PR regimen versus placebo varied: 13% to 74% versus 10% to 68% for *L. major*; 4% versus 3% for *L.chagasi*; and 82% versus 34% for *L.braziliensis*. Overall, any PR regimen was more effective than placebo to achieve clinical cure (RR: 1.49; 95% CI: 1.04–2.13; P = 0.031; heterogeneity *χ^2^* = 22.33, P = 0.002, *I^2^* = 69%). (Figure 2) The RRs (95% CIs) from six trials of the old world CL and from two trials of the new world CL were 1.27 (0.91–1.77; P = 0.165; heterogeneity *χ^2^* = 11.93, P = 0.036, *I^2^* = 58%) and 2.34 (1.48–3.71; P<0.001; heterogeneity *χ^2^* = 0.18, P = 0.668, *I^2^* = 0%), respectively. The meta-regression analysis suggested the type of PR regimen as a main source of heterogeneity (P = 0.024), but neither the duration of lesion nor the length of treatment. The heterogeneity disappeared (*I^2^* = 0%), when the data were pooled according to the type of PR regimen. Topical PR was more effective than placebo when it was combined with MBCL (2.58; 1.76–3.76; P<0.001; heterogeneity *χ^2^* = 0.37, P = 0.830, *I^2^* = 0%) compared to when it was used alone (1.01; 0.87–1.18; P = 0.867; heterogeneity *χ^2^* = 1.82, P = 0.769, *I^2^* = 0%). The results for the secondary outcome were similar (data not shown).

### Efficacy of PR versus SbV

Table 2 summarizes the existing data on efficacy of PR regimen as compared to controls. In the old world CL, the evidence only addressed the comparison between topical PR and intralesional MA. No trials compared topical PR or parenteral PR with parenteral SbV. In the new world CL, various topical or parenteral PR regimens were compared with parenteral SbV, but no trials compared any PR regimens with intralesional SbV.

**Table 2:** Summary of Trials Comparing Paromomycin with Placebo or Antimony Compounds*.

Type of CL	Paromomycin Regimen	Control Group		
		Placebo	Intralesional SbV	Parenteral SbV
Old World CL	Topical PR only	1.01 (0.87, 1.18)[29],[32],[34],[36]	0.70 (0.26, 1.89)[17],[33]	No data
	Topical PR/MBCL	2.99 (1.56, 5.75)[28],[37]	No data	No data
	Parenteral PR	No data	No data	No data
New World CL	Topical PR only	1.30 (0.09, 19.8)[35]	No data	0.68 (0.46, 1.00)[3]
	Topical PR/MBCL	2.39 (1.50, 3.80)[16]	No data	0.68 (0.46, 1.00)[3]
	Topical PR/MBCL/Parenteral SbV	No data	No data	0.65 (0.49, 0.87)[6]
	Parenteral PR	No data	No data	0.88 (0.56, 1.38)[4],[5]

Abbreviations: CL, cutaneous leishmaniasis; SbV, pentavalent antimony compounds (including meglumine antimoniate and sodium stibogluconate); PR, paromomycin; MBCL, methylbenzethonium chloride.

*: Pooled RRs and 95% CIs of clinical cure were calculated using an inverse-variance weighted random-effects model. Pooled RRs greater than 1 indicate that the results favor paromomycin regimen to control regimen.

**Figure 3:**
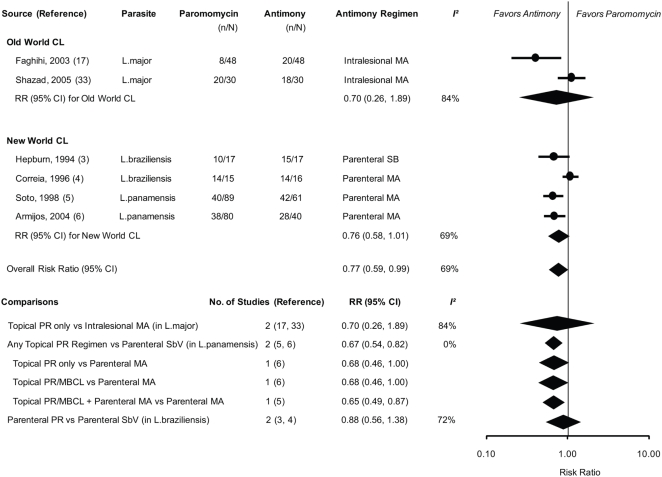
Meta-analysis of the Efficacy of Paromomycin Compared with Pentavalent Antimony Compounds*†. Abbreviations: CI, confidence interval; CL, cutaneous leishmaniasis; MA, meglumine antimoniate; MBCL, methylbenzethonium chloride; PR, paromomycin; RR, risk ratio; SB, sodium stibogluconate. * Pooled RRs and 95% CIs of clinical cure were calculated using an inverse-variance weighted random-effects model and displayed in diamonds in the figure. The *I^2^* statistic describes the percentage of total variation across the studies that is attributable to heterogeneity rather than chance. † Parenteral antimony compounds include parenteral MA and parenteral SB.

Data from SbV-controlled trials were pooled by the type of PR and SbV regimens. Absolute rate of clinical cure comparing various PR regimens versus SbV regimens was the following: 17% to 67% versus 42% to 60% for *L.major*; 59% to 93% versus 88% for *L.braziliensis*; and 45% to 48% versus 69% to 70% for *L. panamensis*. Overall, any PR regimen was less effective than any SbV regimen to achieve clinical cure (0.77; 0.59–0.99; P = 0.043; heterogeneity *χ^2^* = 16.08, P = 0.007, *I^2^* = 69%). (Figure 3) In the old world CL, the efficacy of topical PR was not significantly different from that of intralesional MA (0.70; 0.26–1.89; P = 0.480; heterogeneity *χ^2^* = 6.06, P = 0.014, *I^2^* = 84%). In the new world CL, topical PR was less effective than parenteral MA (0.67; 0.54–0.82; P<0.001; heterogeneity *χ^2^* = 0.03, P = 0.856, *I^2^* = 0%), whereas no significant difference was found between parenteral PR and parenteral SbV (0.88; 0.56–1.38; P = 0.567; heterogeneity *χ^2^* = 3.52, P = 0.061, *I^2^* = 72%). Similar results were observed for the secondary outcome (data not shown).

### Side effects of PR

Only a small number of trials reported extractable data on side effects. (Table 3) In general, local side effects were more common with topical treatment and systemic side effects were more common with parenteral treatment. Local reactions seem to occur more frequently when topical PR was combined with MBCL (1.60; 0.98–2.61; P = 0.061; heterogeneity *χ^2^* = 0.15, P = 0.701, *I^2^* = 0%), as compared to when topical PR was used alone (1.07; 0.52–2.21; P = 0.850; heterogeneity *χ^2^* = 3.36, P = 0.339, *I^2^* = 11%). Systemic side effects were less frequent with topical or parenteral PR as compared to parenteral SbV [3]–[5]. Laboratory data were not available for extraction; however, no significant difference was reported on blood counts, chemistry, and liver function tests between any topical PR regimen and placebo [19]–[22]. Bone marrow suppression and abnormal liver function tests were reported more often with parenteral SbV as compared to parenteral PR [5].

**Table 3:** Side Effects of Paromomycin Compared with Placebo and Antimony Compounds.

Comparisons	Paromomycin (n/N)	Control (n/N)
***Local reactions*** *		
**Topical PR only vs Placebo**		
El-Safi, 1990 [36]	2/20	0/20
Salah, 1995 [34]	6/52	6/56
Asilian, 1995 [32]	8/126	11/125
Iraji, 2005 [29]	3/40	0/40
**RR (95% CI)**	**1.07 (0.52, 2.21)**	*I^2^* = 11%
**Topical PR only vs Intralesional MA**		
Shazad, 2005 [33]	1/30	3/30
**Topical PR only vs Parenteral MA**		
Armijos, 2004 [3] †	13/40	0/40
**Topical PR/MBCL vs Placebo**		
El-On, 1992 [37]	3/40	0/16
Arana, 2001 [16]	22/38	14/38
**RR (95% CI)**	**1.60 (0.98, 2.61)**	*I^2^* = 0%
**Topical PR/MBCL vs Parenteral MA**		
Armijos, 2004 [3] †	7/40	0/40
***Myalgia***		
**Topical PR only vs Parenteral MA**		
Armijos, 2004 [3]	0/40	1/40
**Topical PR/MBCL vs Parenteral MA**		
Armijos, 2004 [3]	0/40	1/40
**Parenteral PR vs Parenteral Antimony Compounds** ‡		
Hepburn, 1994 [5]	1/17	17/17
Correia, 1996 [4]	2/15	8/16
**RR (95% CI)**	**0.16 (0.05, 0.48)**	*I^2^* = 14%
***Generalized Symptoms*** §		
**Topical PR only vs Parenteral MA**		
Armijos, 2004 [3]	2/40	14/40
**Topical PR/MBCL vs Parenteral MA**		
Armijos, 2004 [3]	6/40	14/40
**Parenteral PR vs Parenteral Antimony Compounds** ‡		
Hepburn, 1994 [5]	0/17	3/17
Correia, 1996 [4]	10/15	12/16
**RR (95% CI)**	**0.64 (0.16, 2.54)**	*I^2^* = 33%
***Headache***		
**Topical PR only vs Parenteral MA**		
Armijos, 2004 [3]	1/40	5/40
**Topical PR/MBCL vs Parenteral MA**		
Armijos, 2004 [3]	2/40	5/40
**Parenteral PR vs Parenteral SB**		
Hepburn, 1994 [5]	0/17	3/17
***Arthralgia***		
**Topical PR only vs Parenteral MA**		
Armijos, 2004 [3]	0/40	1/40
**Topical PR/MBCL vs Parenteral MA**		
Armijos, 2004 [3]	0/40	1/40
**Parenteral PR vs Parenteral MA**		
Correia, 1996 [4]	0/15	5/16
***Generalized Eruption***		
**Parenteral PR vs Parenteral SB**		
Hepburn, 1994 [5]	1/17	1/17

Abbreviations: PR, paromomycin; RR, risk ratio; CI, confidence interval; MBCL, methylbenzethonium chloride; MA, meglumine antimoniate; SB, sodium stibogluconate.

*: Local reactions include pain, burning sensation, pruritus, erythema, edema, and inflammation at the site of administration.

**†:** Armijos et al [3] reported the number of subjects who experienced each category of local side effects, without providing a cumulative number of subjects. Therefore, the number of those who developed local inflammation was presented for local reaction.

**‡:** Parenteral antimony compounds include parenteral MA and parenteral SB.

**§:** Generalized symptoms include fever, malaise, weakness, and anorexia.

### Sensitivity analyses and publication bias

**Figure 4:**
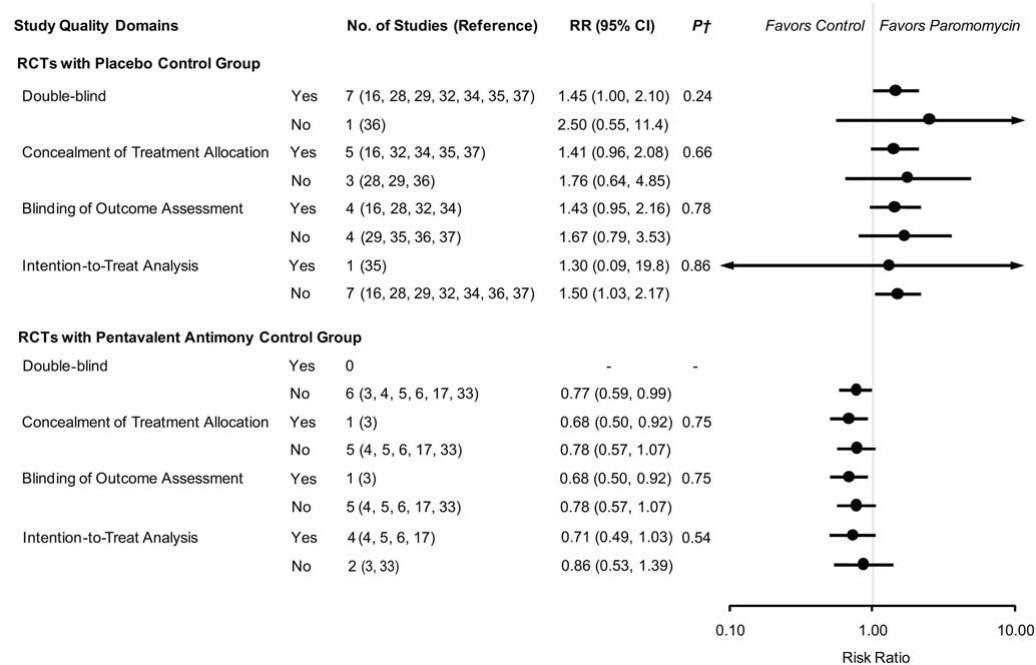
Influence of Study Quality Criteria on Pooled Estimates*. * Pooled RRs and 95% CIs of clinical cure were calculated by study quality components, using an inverse-variance weighted random-effects model. † Adjusted for paromomycin regimen among trials with placebo control group and for antimony regimen among trials with pentavalent antimony compound control group.

Concealment of treatment allocation was adequate in six trials [3],[16],[32],[34],[35],[37] and outcome assessment was blinded in five trials [3],[16],[28],[32],[34]. The intention-to-treat analysis was performed in five trials [4]–[6],[17],[35]. Trials that did not meet the study quality criteria tended to slightly exaggerate the efficacy of PR as compared to placebo or SbV. (Figure 4) In addition, the pooled estimates were not significantly changed when an individual trial was omitted. There was no evidence of publication bias based on the Begg's test and Egger's test (P = 0.621 and P = 0.126, respectively, for placebo-controlled trials; and P = 0.348 and P = 0.242, respectively, for SbV-controlled trials).

## Discussion

In our meta-analysis, we found that topical PR showed therapeutic activity only when it was combined with MBCL. Local reactions also tended to increase with MBCL. The efficacy of topical PR did not differ from that of intralesional SbV in the old world CL, whereas its efficacy was inferior to parenteral SbV in the new world CL. No significant difference was found between parenteral PR and SbV in the new world CL. However, due to small number and heterogeneous quality of included studies, our findings should be interpreted with caution.

### Randomized controlled trials with placebo control group

Our analysis suggested that topical PR with MBCL showed therapeutic activity, whereas topical PR with soft paraffin or urea did not. Our finding is further supported by several lines of experimental evidence. MBCL, a quaternary ammonium compound, suppresses the growth of *L.major* in an *in vitro* model and increases cutaneous permeability of PR [11],[38]. *In vivo* studies suggested the synergistic action between PR and MBCL [12],[39]. In a randomized controlled study comparing topical PR with MBCL, topical PR with urea, and parenteral MA [3], Armijos et al. found a non-significant higher cure rate in 12% MBCL group than in 10% urea group (79.3% vs. 70.0%). But the study was underpowered for the comparison between the two topical regimens. We also found that local reactions appeared to increase when topical PR was combined with MBCL. It is not clear whether a lower MBCL concentration (i.e., 5% vs. 12%) can reduce local reactions without compromising efficacy. El-On et al. compared 5% and 12% MBCL as an adjunct to topical PR, and found cure rates of 66.6% and 76.6%, respectively [37]. Severe local reactions were observed only in patients treated with 12% MBCL. However, other characteristics of topical formulas, including the composition of vehicle [40],[41] and application methods, such as occlusion [42], also play key roles in determining the efficacy.

Although the type of topical PR was responsible for the heterogeneity among placebo-controlled trials, other important clinical factors, such as differences in parasite species and their clinical manifestation (i.e. self-cure rate and types of lesions), length of treatment, and duration of the lesions should be considered for several reasons. The tendency for spontaneous cure or progression to a more severe form of CL varies among the species. Even in the old world CL, spontaneous cure rate at 3 months is 60–70% for *L.major*, but <1% for *L.tropica* [8]. Moreover, *L.braziliensis* infection is associated with a more severe and prolonged course, a higher risk of progression to mucocutaneous form [8], and a lower self-healing rate [43]. Among placebo-controlled trials included in our meta-analysis, the clinical cure rate varied by species: up to 68% for *L.major*, 3% for *L.chagasi*, and 34% for *L.braziliensis*. Limited *in vitro* and *in vivo* observations also suggested that the new world CL was more refractory to PR than the old world CL [15],[44]. Although an *in vitro* [39] and several human studies [3],[16],[43],[45] of topical PR with MBCL demonstrated its efficacy against the new world CL, most clinicians do not use local treatments for *L.braziliensis* complex infection. Another important characteristic to be considered is the type of lesions. Depending on the stages of infection and species, lesions can vary from small erythema to nodular or ulcerative lesions [8]. Ulcerated lesions are typical of *L.major* and the new world species, whereas nodular lesions are typical of *L.aethiopica* and *L.donovani* and hyperkeratotic lesions of *L.tropica*. Topical agents may have better absorption in ulcerative lesions than in nodular lesions. Such differences in clinical features were not well-reflected in our analysis due to the limited data and a small number of trials.

Lack of significant association between the length of treatment and its efficacy in meta-regression analysis does not necessarily exclude the benefit of a longer treatment course. In fact, none of the included trials involved a direct comparison. Asilian et al. randomly assigned patients with CL caused by *L.major* to either two-week or four-week PR treatment, and found a significantly better cure rate and reduced need for SbV rescue treatment in the four-week group [22]. Even a small improvement in cure rate can lead to considerable benefit to patients by avoiding serious systemic side effects by SbV treatment.

In a self-healing disease like CL, the duration of lesions may have a crucial role on cure rate. Insufficient number and inadequate reporting [6], [17], [34]–[36] of included trials did not allow enough power to detect the trend, but it is possible that the efficacy of PR may diminish, as the lesions get older.

### Randomized controlled trials with SbV control group

An inadequate number of trials did not allow us to examine the efficacy of various PR and SbV regimens for each parasite species. No trials compared the combination of topical PR and MBCL with intralesional or parenteral SbV in the old world CL; or topical PR and intralesional SbV in the new world CL. In general, the old world CL is treated with intralesional SbV, whereas the new world CL is treated with parenteral SbV due to high risk of mucocutaneous involvement [8]. Our study suggests that topical and parenteral PR have lower side effects as compared to intralesional and parenteral SbV.

### Limitations

A major limitation to our study is the small number of included trials. There were several comparisons that were based on only two to three trials. This increases the uncertainty of pooled estimates. Certain species, such as *L.tropica*, have not been examined in the included trials. These limit generalizability of our findings. In addition, overall poor quality in conducting and reporting trials was noted. El-On et al. [37] used a cross-over design which is less desirable in assessing the efficacy of a treatment in a self-limited disease, as criticized in a recent review [46]. Inadequate reporting of demographic characteristics of participants [3],[5],[6],[16],[35],[36], parasite species [3],[17],[29], duration of lesions [6], [17], [34]–[36], and quantitative data on side effects [6],[17],[18],[24],[27],[28] were very common. Standardization of study protocols has been suggested to facilitate between-study comparisons [47]. Furthermore, in our sensitivity analysis, trials that did not meet the study quality criteria tended to slightly exaggerate the efficacy of PR compared with control group. For unbiased and reliable evaluation, investigators should address appropriate quality criteria in design and conduct of trials and strictly follow the reporting standards such as CONSORT [48],[49]. Finally, publication bias cannot be excluded reliably in our meta-analysis, because of low sensitivity of the Begg's test and Egger's test in meta-analyses of fewer than 20 trials [50]. However, it has been reported that publication bias did not change the conclusions in most cases [51].

### Implications

The main findings of our meta-analysis can be summarized as the following: 1) topical PR appears to demonstrate therapeutic activity against the old world and new world CL, with a tendency of increased local reactions, when it was combined with MBCL; 2) in the old world CL, the efficacy of topical PR is not different from that of intralesional SbV; and 3) in the new world CL, the efficacy of topical PR is inferior to that of parenteral SbV, whereas the efficacy of parenteral PR is not different from that of parenteral SbV. Although similar findings have been described in the past [8],[16],[22],[52], a valuable contribution of our meta-analysis is to provide their quantitative dimension. For clinicians, this meta-analysis confirms that the existing evidence does not support topical PR as an acceptable treatment of the new world CL. However, topical PR with MBCL could be a therapeutic alternative for selected cases of old world CL with lower risk of mucocutaneous involvement, due to its lack of serious systemic side effects.

An acceptable alternative should demonstrate efficacy as well as local tolerability to ensure compliance. Sustained availability is also an issue. To this end, the efforts are currently made to develop formulations that has equivalent efficacy to that of first-generation formulations and local side effect profile similar to that of second-generation formulations [52]. For instance, a few randomized controlled trials evaluating a new topical PR-based formulation, WR 279396, compared to placebo or pure topical PR in CL caused by *L.major* are under way. This new formation was found to have therapeutic activity as well as cosmetic effects in an animal model [41]. Future research on topical PR in treatment of the old world CL merits addressing the following issues: examining the efficacy of various topical PR regimens in other species, such as *L.tropica*; comparison between topical PR with MBCL and topical PR only; evaluation of topical PR with different MBCL concentration for their efficacy and tolerability; and development of new formulations that has similar or superior efficacy and better tolerability than topical PR and MBCL.

## Supporting Information

Appendix S1 Search Strategy
(0.03 MB DOC)

Checklist S1 QUOROM Checklist
(0.18 MB PDF)
