# Supplementary material for: Is Paromomycin an Effective and Safe Treatment against Cutaneous Leishmaniasis? A Meta-Analysis of 14 Randomized Controlled Trials
Source: PLoS Negl Trop Dis. 2009 Feb 17;3(2):e381. doi: 10.1371/journal.pntd.0000381 (PMC2637543; doi:10.1371/journal.pntd.0000381)
Supplement: Appendix S1 — Search Strategy (0.03 MB DOC) [file pntd.0000381.s001.doc]

**APPENDIX**

The following strategies were used to identify all randomized controlled trials that were conducted in humans and evaluated the efficacy or safety of paromomycin treatment in CL. Initial search was performed on June 30, 2007 and updated on August 31, 2007.

**A. PubMed:** 23 reports were identified.

((cutaneous leishmaniasis[tiab] OR cutaneous leishmaniasis[mh]) AND (paromomycin[tiab] OR paromomycin[mh] OR aminosidine[tiab])) AND ((“randomized controlled trial”[PT] OR “controlled clinical trial”[PT] OR “randomized controlled trials”[MH] OR “random allocation”[MH] OR “double-blind method”[MH] OR “single-blind method”[MH]) NOT (animal[MH] NOT human[MH])) NOT (review[PT] OR meta-analysis[PT])

**B. Scopus:** 44 reports were identified.

TITLE-ABS-KEY(cutaneous leishmaniasis AND (paromomycin OR aminosidine)) AND ALL("randomized controlled trial" OR "controlled clinical trial" OR "randomization" OR "double blind procedure" OR "single blind procedure" OR "randomized" OR "randomised" OR "random" OR "randomly") AND (EXCLUDE(DOCTYPE, "re"))

**C. Cochrane Central Register of Clinical Trials:** 27 reports were identified.

((cutaneous leishmaniasis):ti,ab,kw) AND (((paromomycin):ti,ab,kw) OR ((aminosidine):ti,ab,kw))
