# Supplementary material for: Is Paromomycin an Effective and Safe Treatment against Cutaneous Leishmaniasis? A Meta-Analysis of 14 Randomized Controlled Trials
Source: PLoS Negl Trop Dis. 2009 Feb 17;3(2):e381. doi: 10.1371/journal.pntd.0000381 (PMC2637543; doi:10.1371/journal.pntd.0000381)
Supplement: Checklist S1 — QUOROM Checklist (0.18 MB PDF) [file pntd.0000381.s002.pdf]

Improving the quality of reports of meta-analyses of randomized controlled trials: the QUOROM statement checklist

Heading	Subheading	Descriptor	Reported? (Y/N)	Page number
Title		Identify the report as a meta-analysis [or systematic review] of RCTs[26]	Yes	1
Abstract		Use a structured format[27]	Yes	2
		**Describe**		
	Objectives	The clinical question explicitly	Yes	2
	Data sources	The databases (ie, list) and other information sources	Yes	2
	Review methods	The selection criteria (ie, population, intervention, outcome, and study design); methods for validity assessment, data abstraction, and study characteristics, and quantitative data synthesis in sufficient detail to permit replication	Yes	2
	Results	Characteristics of the RCTs included and excluded; qualitative and quantitative findings (ie, point estimates and confidence intervals); and subgroup analyses	Yes	2
	Conclusion	The main results	Yes	2
		**Describe**		
Introduction		The explicit clinical problem, biological rationale for the intervention, and rationale for review	Yes	3
Methods	Searching	The information sources, in detail[28] (eg, databases, registers, personal files, expert informants, agencies, hand-searching), and any restrictions (years considered, publication status,[29] language of publication[30,31])	Yes	4, 23
	Selection	The inclusion and exclusion criteria (defining population, intervention, principal outcomes, and study design[32])	Yes	4
	Validity assessment	The criteria and process used (eg, masked conditions, quality assessment, and their findings[33–36])	Yes	5
	Data abstraction	The process or processes used (eg, completed independently, in duplicate)[35,36]	Yes	5
	Study characteristics	The type of study design, participants' characteristics, details of intervention, outcome definitions, &c,[37] and how clinical heterogeneity was assessed	Yes	5, 6
	Quantitative data synthesis	The principal measures of effect (eg, relative risk), method of combining results (statistical testing and confidence intervals), handling of missing data; how statistical heterogeneity was assessed;[38] a rationale for any a-priori sensitivity and subgroup analyses; and any assessment of publication bias[39]	Yes	5, 6
Results	Trial flow	Provide a meta-analysis profile summarising trial flow (see figure)	Yes	7, Figure 1
	Study characteristics	Present descriptive data for each trial (eg, age, sample size, intervention, dose, duration, follow-up period)	Yes	7, Table 1
	Quantitative data synthesis	Report agreement on the selection and validity assessment; present simple summary results (for each treatment group in each trial, for each primary outcome); present data needed to calculate effect sizes and confidence intervals in intention-to-treat analyses (eg 2 $\times$2 tables of counts, means and SDs, proportions)	Yes	7-9, Figures 2-4, Tables 2, 3
Discussion		Summarise key findings; discuss clinical inferences based on internal and external validity; interpret the results in light of the totality of available evidence; describe potential biases in the review process (eg, publication bias); and suggest a future research agenda	Yes	10-14

Quality of reporting of meta-analyses
